# Enhanced photoelectrochemical activities for water oxidation and phenol degradation on WO_3_ nanoplates by transferring electrons and trapping holes

**DOI:** 10.1038/s41598-017-01300-7

**Published:** 2017-05-02

**Authors:** Liqun Sun, Yuying Wang, Fazal Raziq, Yang Qu, Linlu Bai, Liqiang Jing

**Affiliations:** 1 Key Laboratory of Functional Inorganic Materials Chemistry (Heilongjiang University), Ministry of Education, International Joint Research Center for Catalytic Technology, School of Chemistry and Materials Science, Harbin, 150080 P.R. China; 2 College of Chemical Engineering, Daqing Normal University, Key Laboratory of Oilfield Applied chemistry, College of Heilongjiang Province, Daqing, 163712 P.R. China; 3 College of Chemical and Environmental Engineering, Harbin University of Science and Technology, Harbin, 150040 P.R. China

## Abstract

It is highly desired to improve the photoelectrochemical (PEC) performance of nanosized WO_3_ by artificially modulating the photogenerated electrons and holes simultaneously. Herein, WO_3_ nanoplates have been successfully prepared by a simple one-pot two-phase separated hydrolysis-solvothermal method, and then co-modified with RGO and phosphate acid successively by wet chemical processes. Subsequently, the as-prepared WO_3_-based nanoplates were immobilized on the conductive glasses to explore the PEC activities for both water oxidation to evolve O_2_ and phenol degradation. It is clearly demonstrated that the co-modified WO_3_ nanoplates exhibit significantly improved PEC activities compared with pristine WO_3_, especially for that with the amount-optimized modifiers by ca. 6-time enhancement. Mainly based on the evaluated hydroxyl radical amounts produced and the electrochemical impedance spectra, it is suggested that the improved PEC activities are attributed to the greatly enhanced photogenerated charge separation after chemically modification with RGO and phosphate groups to WO_3_, respectively by transferring electrons as the collectors and trapping holes via the formed negative field after phosphate disassociation. This work provides a feasible synthetic strategy to improve the photoactivities of nanosized WO_3_ for energy production and environmental remediation.

## Introduction

The increasing energy crisis urges the seeking of novel energy sources to realize the useful chemical conversion. Synergistically utilizing the photo and electricity as the clean energy sources, the PEC techniques exhibit significant advantages in the efficient chemical conversion^1–3^, hence regarded as one of the most promising catalytic techniques for splitting water and degradation of environmental pollutants^4–6^. The efficiency of the PEC techniques is directly associated with the photocatalytic activity of the photoelectrode material. Noteworthily, to adopt and design the suitable semiconductor material as the photoelectrode is the key to improve the PEC techniques.

Tungsten oxide (WO_3_) is comprehensively applied as the photoanode material in PEC devices because of its favourable valance band (VB) edge position for O_2_ evolution (3.0 V versus the normal hydrogen electrode, NHE), abundance and low cost^7–10^. However, for WO_3_ the sluggish kinetics of holes, slow charge transfer at the semiconductor/electrolyte interface and fast electron-hole recombination in both bulk and surface substantially all limit its application as efficient photoanode material^11^. Therefore, it is highly desired to enhance the separation of photogenerated charges to improve the photocatalytic efficiency of WO_3_.

Various methods have been developed to improve the photocatalytic efficiency by modifying the semiconductor, which include coupling with other semiconductors^12, 13^, noble metals^14, 15^ and graphene-based materials^16, 17^ as well as doping with metal ions^18–20^ and nonmetals^21^. Among all the materials applied for modification, due to the advantageous electronic and physicochemical properties, reduced graphene oxide (RGO) has been widely applied to function as the photogenerated electrons shuttle to facilitate the photogenerated charge seperation^22–25^. Although some works have been reported on the improved photocatalytic activities of semiconductors by coupling RGO^26, 27^, however the photogenerated charge transfer and separation mechanism related to RGO modified WO_3_ as photocatalyst is still ambiguous currently. Therefore, it is significant to modify WO_3_ with RGO to enhance the PEC performances and to investigate the detailed process mechanism.

Besides transporting electrons to the modifier, to speed the transport of photogenerated holes to the surface of semiconductor becomes another feasible strategy to further benefit the effective photogenerated charge separation. Both the photocatalytic water splitting to evolve O_2_ and the pollutant degradation are involved with the hydroxyl radicals originating from the oxidation of holes with H_2_O. Therefore, it’s desirable to increase the amount of holes on the photocatalyst surface to facilitate the production of hydroxyl radicals for efficient PEC process^28, 29^. As reported the phosphate modification of semiconductor could form the negative field on the semiconductor surface so as to trap holes resulting in the effective separation of photogenerated charge separation^30, 31^. Naturally expected, the phosphate modification also applies to WO_3_ by trapping holes to facilitate the PEC water oxidation as well as the pollutant degradation, however, which has never been reported yet and relevant mechanism is unclear. Hence, it is also meaningful to improve the PEC performance of WO_3_ by phosphate modification to trap holes and study the detailed process mechanism.

In general, it is a common idea to enhance the photogenerated charge separation by controlling electrons or holes for efficient photocatalysis. While obviously to modulate electrons and holes simultaneously is more efficient and of great significance, which has not been reported as far as we know. Herein, it is well demonstrated that the PEC activities for water oxidation and pollutant degradation on WO_3_ nanoplates could be obviously improved by chemical co-modification with RGO and phosphate groups. Noteworthily, it is clearly confirmed that the improved photocatalytic activities be attributed to the greatly enhanced photogenerated charge separation respectively by transferring electrons with RGO as the collectors and trapping holes via the formed negative field after phosphate disassociation. This work would provide an advantageous design strategy to obtain high-performance WO_3_-based photoanodes for vital PEC reactions.

## Results

### Structural characterization and surface composition

The crystalline structures of as-synthesized samples were investigated by X-ray diffraction (XRD) spectrometer as shown in Figure S1. For WO_3_, the peaks at 23.1, 23.6, 24.4, 49.9 and 56.0°, which can be assigned to the monoclinic tungsten oxide of the (002), (020), (200), (140) and (420) plane respectively (JCPDS Cards No. 43-1035)^32^, confirming the successful synthesis of WO_3_ by the hydrolysis-solvothermal method. The samples xRGO/WO_3_, WO_3_-yP and 2RGO/WO_3_-yP all comprise crystalline monoclinic tungsten oxide, indicating that the crystalline phase of the tungsten oxide nanostructure has been preserved after the introduction of RGO and phosphate. In addition, for RGO involved samples xRGO/WO_3_ and 2RGO/WO_3_-yP, no characteristic peaks assigned to RGO were detected which might be due to the tiny amount of RGO as well as its poor crystallinity.

**Figure 1:**
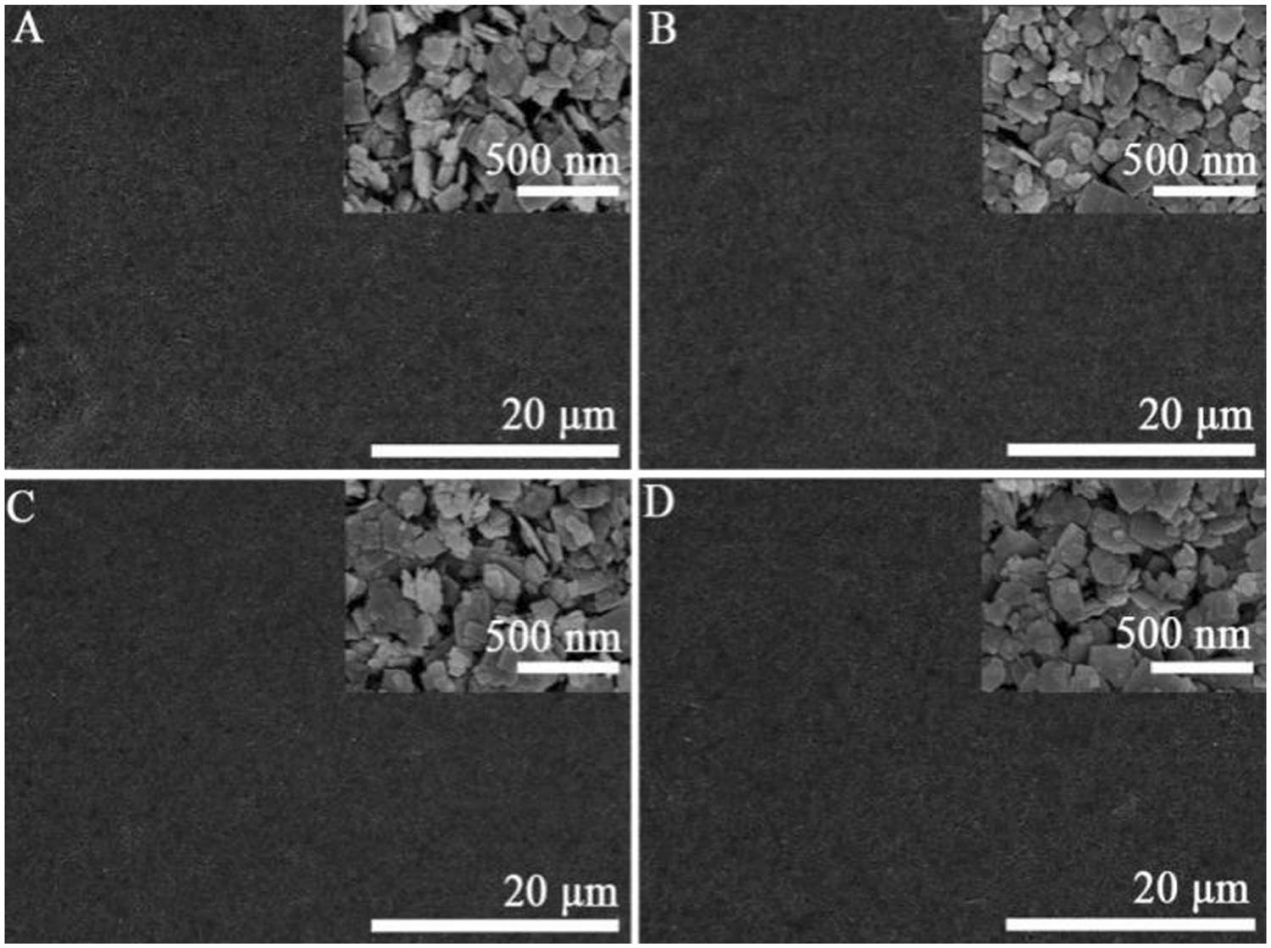
SEM images of WO_3_ (**A**), 2RGO/WO_3_ (**B**), WO_3_-3P (**C**) and 2RGO/WO_3_-3P (**D**).

To examine the optical absorption behavior of different samples, the diffuse reflectance spectra (DRS) of the samples have been recorded. Figure S2 shows that neither the single-component modification with RGO or phosphate nor the simultaneous modifications with RGO and phosphate would affect the band gap of WO_3_. Moreover, the SEM characterizations were performed to investigate the morphologies of the samples (Fig. 1). The pristine WO_3_ took on the aggregates of nanoplates with the thickness of ca. 25 nm and the length of ca. 200 nm. For the PEC test, the WO_3_-based nanocomposites were fabricated to be thin films dispersed on the FTO coated glasses as the photoanode. The relevant side-viewed SEM images (Figure S3) indicated that comparing with the WO_3_ film, the thickness of the co-modified WO_3_ film with RGO and phosphate kept the same at ca. 2 μm. Similarly, in agreement with the XRD and DRS results, after the modifications the morphologies of WO_3_ nanoplates do not change obviously, which might be due to the tiny modification amount of RGO and phosphate chemically absorbed on the WO_3_ surface.

**Figure 2:**
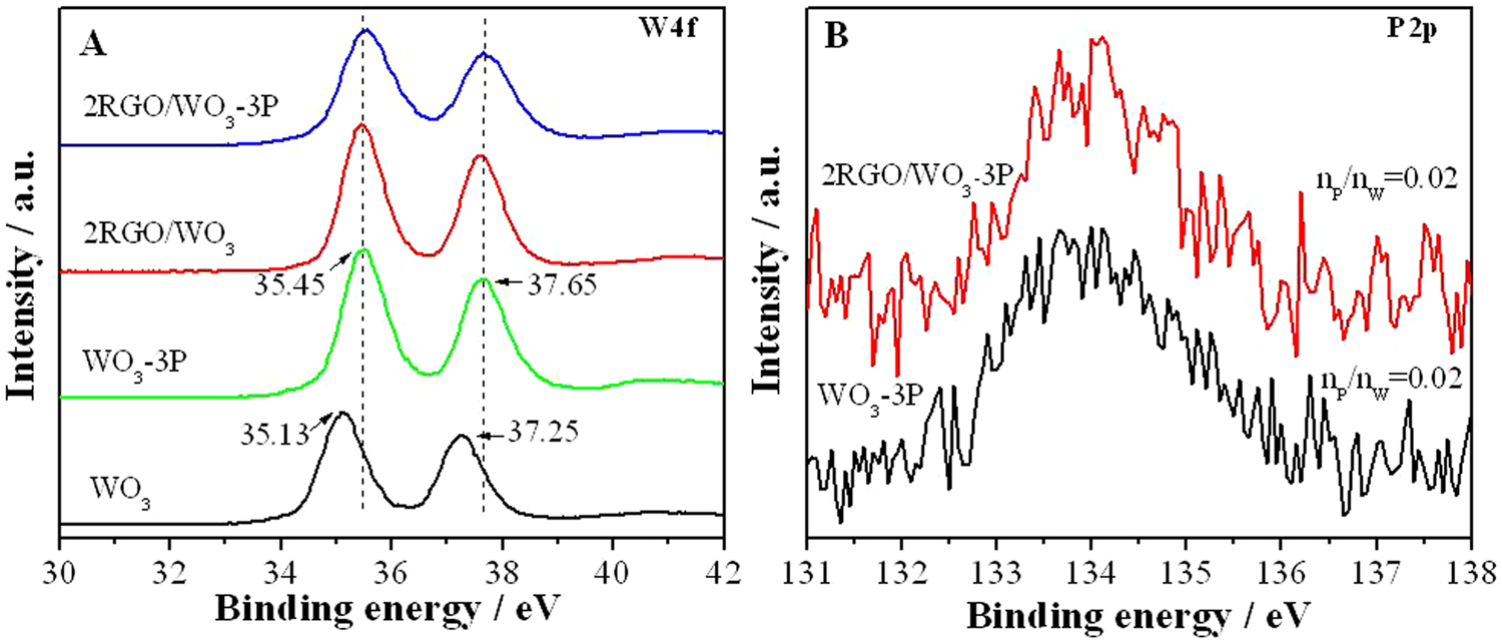
W4f XPS spectra of WO_3_, WO_3_-3P and 2RGO/WO_3_-3P (**A**), P2p XPS spectra of WO_3_-3P and 2RGO/WO_3_-3P (**B**).

To confirm the successful introduction of the two modifiers RGO and phosphate as well as further investigate the chemical states of the components, Raman and XPS characterizations were also performed, respectively. As shown in Figure S4, the Raman peaks were observed at 1360 cm^−1^ and at 1595 cm^−1^ corresponding to D band and G band of RGO, respectively, indicating the existence of small amount RGO^33^. In Fig. 2A, it’s shown that with the modification of RGO, two W4f peaks for 2RGO/WO_3_ both shift to larger binding energies, implying the interaction between RGO and WO_3_. Figures 2B and S5A illustrate the P2p peaks at 133.8 eV verifying the successful introduction of phosphate in the WO_3_-3P and 2RGO/WO_3_-3P hybrid nanocomposites^34^. According to the XPS data that the molar ratios between P and W (n_P_/n_W_) for WO_3_-3P and 2RGO/WO_3_-3P are both 0.02, as marked in Fig. 2B. In addition, as the amount of the phosphate introduced increases, the corresponding P2p peak intensity also increases. Combining with Fig. 2A, it could be concluded that for WO_3_-3P and 2RGO/WO_3_-3P phosphate as modifier also leads to the shift of two W4f peaks to higher binding energies, evidencing the interaction between phosphate and WO_3_. Basing on the characterization results above, the modifiers RGO and phosphate have been proved to be introduced in the WO_3_-based nanocomposites successfully.

### PEC activities for water oxidation and pollutant degradation

**Figure 3:**
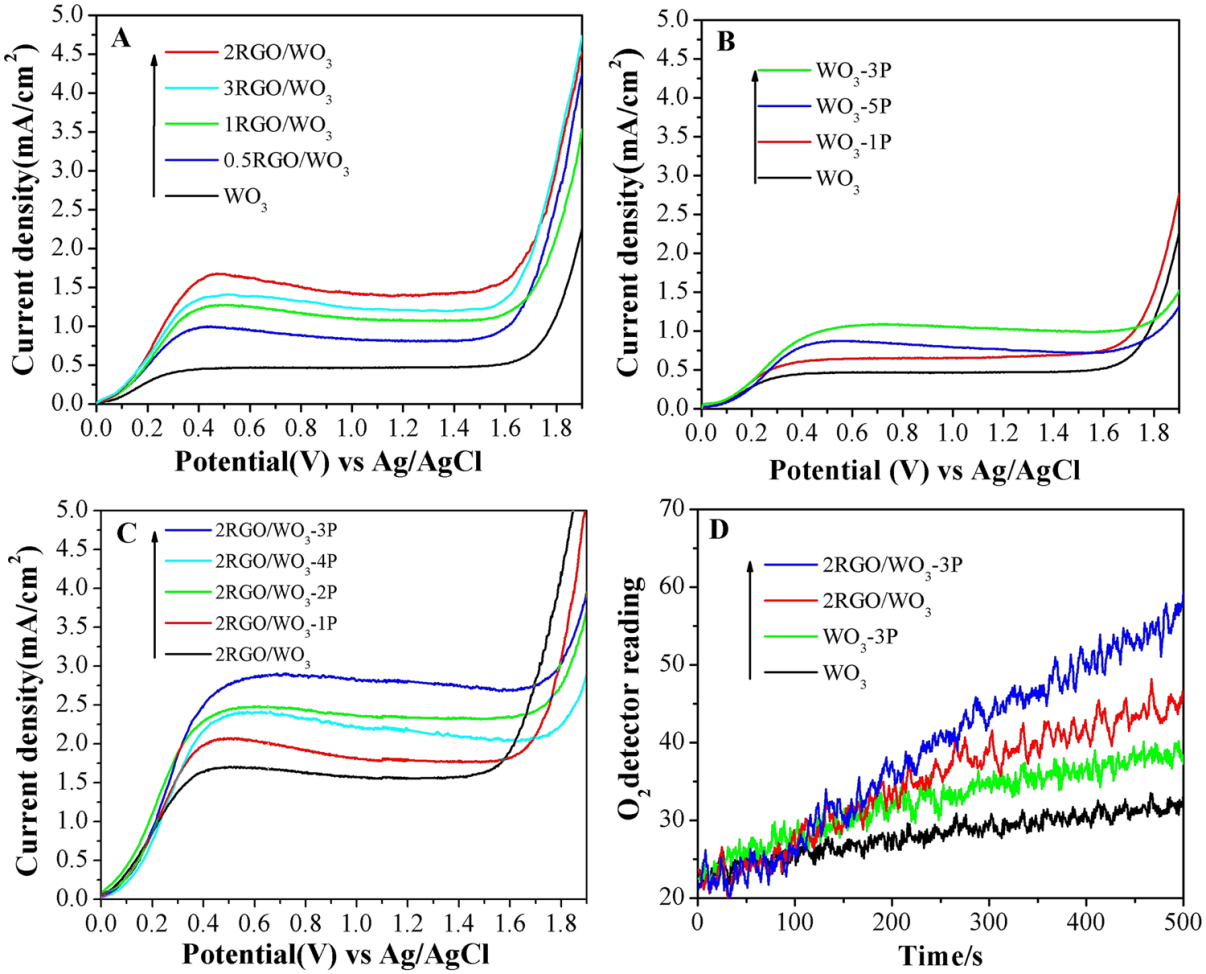
I-V curves of xRGO/WO_3_ films (**A**), WO_3_-yP (**B**) and 2RGO/WO_3_-yP (**C**) under light irradiation, and PEC production curves of O_2_ on WO_3_, WO_3_-3P, 2RGO/WO_3_ and 2RGO/WO_3_-3P (**D**). Potentials were measured in 0.5 M Na_2_SO_4_ electrolyte solution. A three-electrode cell was used with the testing film as the working electrode, Ag/AgCl (saturated KCl solution) as the reference electrode, and Pt plate as the counter electrode.

As Figures S6 and 3A–C the I-V curves illustrated the (photo) current densities for the WO_3_-based photocatalysts under corresponding reaction conditions. Figure S6 shows that the dark scan displayed almost negligible current densities for all samples below 1.4 V, indicating that neither the modifier RGO nor phosphate acts as cocatalyst for the electrochemical water oxidation. As Fig. 3A–C, with the increasing amount of RGO as modifier the photocurrent density increased and the largest photocurrent density was obtained for 2RGO/WO_3_. However the photocurrent density damped when further increasing the RGO amount. Similarly WO_3_-3P with the optimal amount of phosphate exhibited the largest photocurrent density for all phosphate modified WO_3_ samples. With the simultaneous modification of RGO and phosphate, 2RGO/WO_3_-3P exhibited the largest photocurrent density as 2.85 mA/cm^2^ at 0.6 V vs Ag/AgCl, over 6-fold enhancement comparing with pristine WO_3_. Normally the value of photocurrent density could indicate the photogenerated charge separation under light irradiation, which directly affects the PEC performance of the photocatalyst.

As a result, it is naturally anticipated that the amount of evolved O_2_ in the PEC water oxidation utilizing modified WO_3_-based photocatalysts would be remarkably improved. One can see from Fig. 3D that the evolved O_2_ amount of WO_3_ was rather limited. On the contrast, the O_2_ amount for 2RGO/WO_3_ nearly doubled that of the WO_3_. Moreover, it’s worthy noting that the highest evolved O_2_ amount was obtained by 2RGO/WO_3_-P, which is about 3-fold improvement compared with WO_3_. Hence, the large photocurrent density corresponds to the large detector reading for produced O_2_ in the PEC water oxidation, which indicates the photocatalyst with improved charge separation would exhibit more favorable PEC performance in the water oxidation to evolve O_2_.

**Figure 4:**
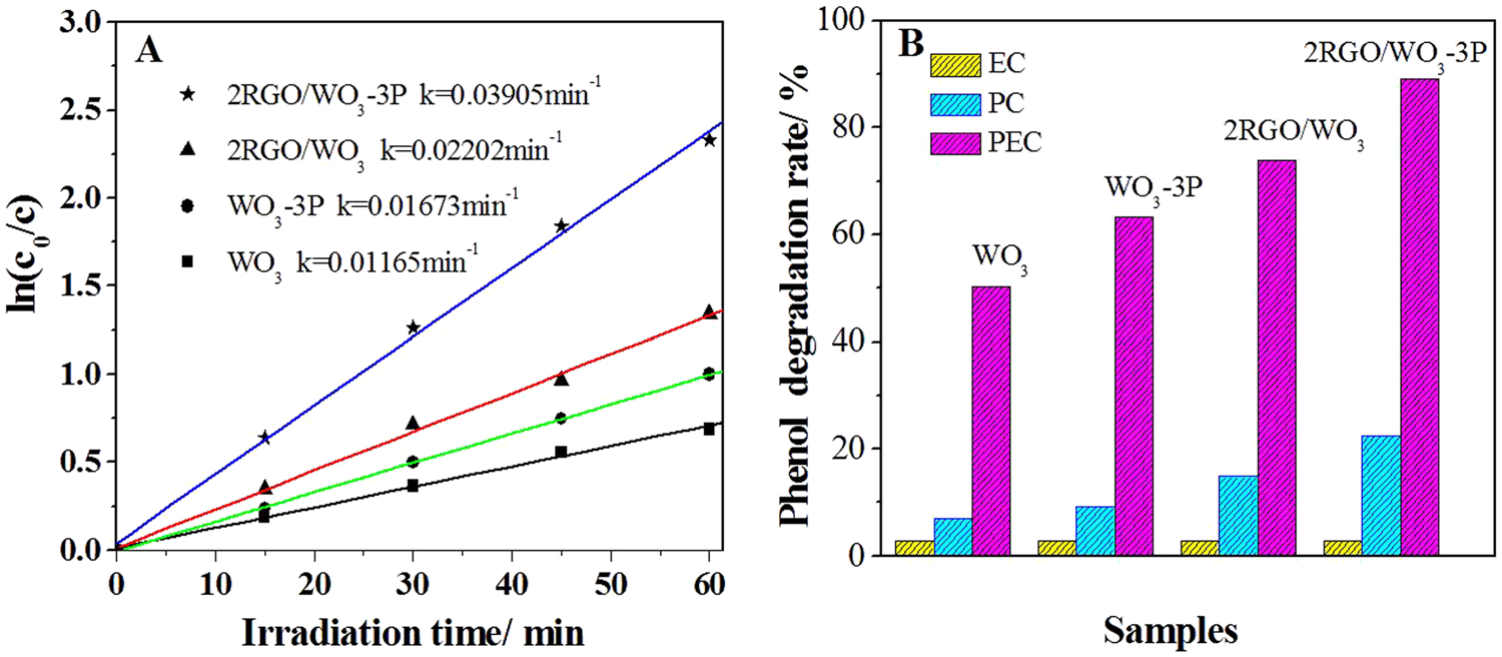
The degradation kinetic plots and corresponding rate constants of WO_3_, WO_3_-3P, 2RGO/WO_3_ and 2RGO/WO_3_-3P for phenol degradation (**A**), and degradation rates of phenol in electrochemical (EC), photochemical (PC) and PEC systems (**B**).

Similar results were also obtained in PEC degradation of phenol. Figure 4A displays degradation kinetic plots for WO_3_, 2RGO/WO_3_, WO_3_-3P and 2RGO/WO_3_-3P. It is obvious that WO_3_ exhibits low photocatalytic activity and the degradation kinetic rate of WO_3_ could be greatly increased after coupling RGO and phosphate. The co-modified sample 2RGO/WO_3_-3P undoubtedly exhibits the highest degradation kinetic rate by nearly 4-time enhancement respectively, compared with the bare WO_3_. As supplementary shown in Figure S5B, the electrochemical (EC), photochemical (PC) and PEC degradation activities of WO_3_, WO_3_-3P, 2RGO/WO_3_ and 2RGO/WO_3_-3P films were also measured. For all samples, the EC performances rival and are rather limited. While under the PC conditions, the modified WO_3_ samples 2RGO/WO_3_, WO_3_-3P and 2RGO/WO_3_-3P all showed improved PC performances, especially 2RGO/WO_3_-3P.

Combining the PEC performances of all samples, it’s could be confirmed that the enhanced PEC performances of modified WO_3_ based photocatalysts in phenol degradation be also ascribed to the improved photocatalytic activities. For 2RGO/WO_3_-3P, it was found that the removal of phenol reached about 89.2% in PEC system after 60 min of reaction. This value was much higher than that in EC system (4.8%) or PC system (20.5%), which further highlights the advantage of PEC technique through the synergy of photo and electricity. The PEC experiments results indicate that the simultaneous modifications of WO_3_ with RGO and phosphate have significantly improved the PEC activities for the water oxidations to evolve O_2_ and pollutant degradation.

## Discussion

On the basis of the above results on the PEC water oxidations and pollutant degradation, the modified WO_3_-based catalysts especially 2RGO/WO_3_-3P with improved photogenerated charge separation, have showed the enhanced photocatalytic performances compared to pristine WO_3_.

**Figure 5:**
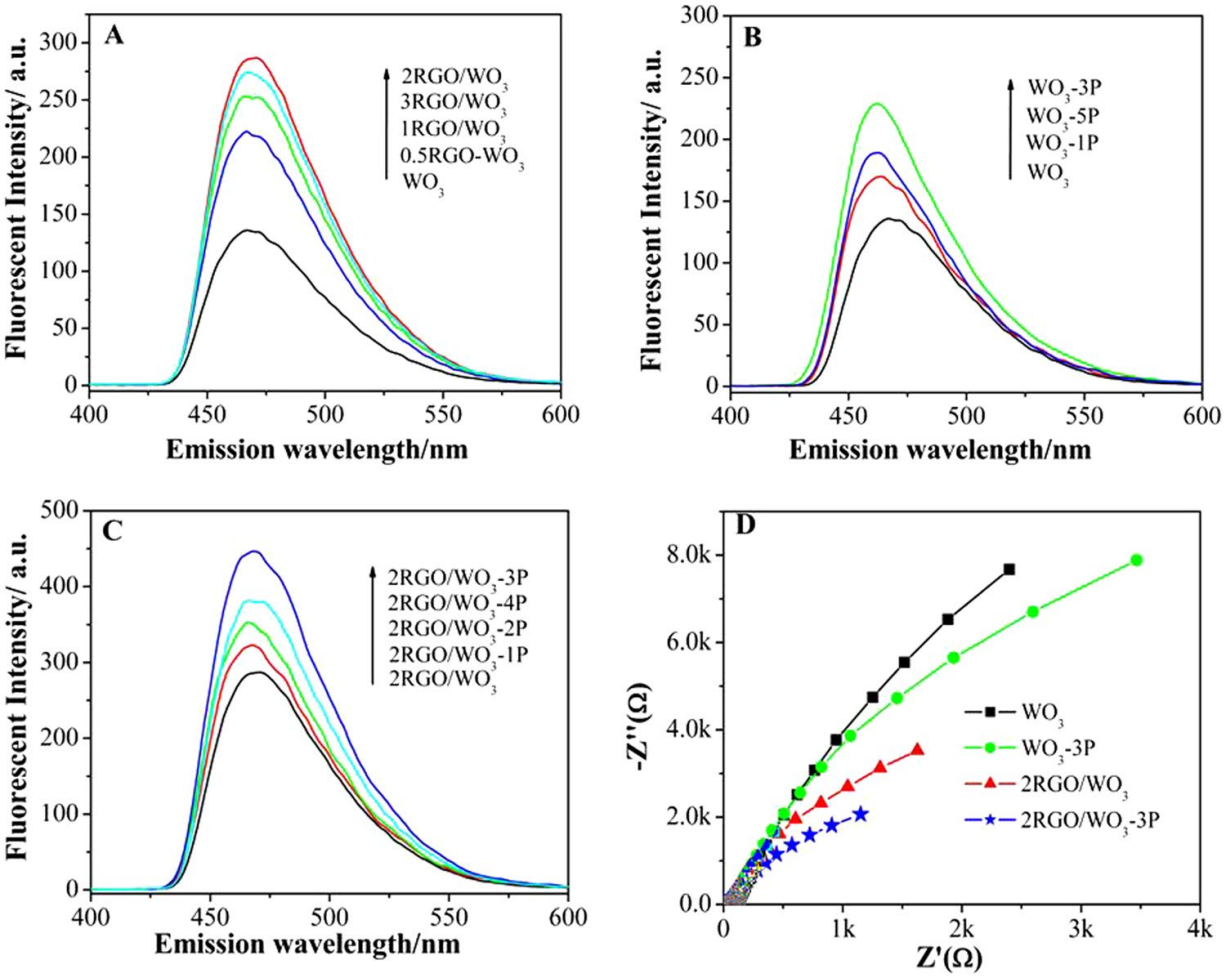
Fluorescent spectra related to the formed hydroxyl radical amount of WO_3_, xRGO/WO_3_ (**A**), WO_3_-yP (**B**), 2RGO/WO_3_-yP (**C**), and electrochemical impedance spectra of WO_3_, WO_3_-3P, 2RGO/WO_3_ and 2RGO/WO_3_-3P (**D**).

To further reveal the improved PEC activities catalyzed by the modified WO_3_ catalysts, the reaction process especially the key intermediate involved in both water oxidation and pollutant degradation should also been considered. As reported the hydroxyl radicals (·OH) are recognized to be important intermediates for both PEC water oxidation and pollutant degradation in the presence of O_2_. The production of ·OH is normally ascribed to be through the reaction between the photogenerated holes and H_2_O so the amount of ·OH produced during the reaction directly affects the photocatalytic activity. Herein the coumarin fluorescent method was used to detect the amount of produced ·OH. Generally, the fluorescent signal is directly proportional to the produced ·OH amount. The amounts of produced ·OH for WO_3_, xRGO/WO_3_, WO_3_-yP and 2RGO/WO_3_-yP are showed in Fig. 5A–C. Pure WO_3_ produces a low amount of ·OH, while for xRGO/WO_3_ samples, the produced ·OH amounts are considerably higher, especially for 2RGO/WO_3_. Moreover, after only coupling phosphate, the produced ·OH amounts are also enhanced and much obvious for WO_3_-3P. Interestingly, the produced ·OH amounts are remarkably increased after co-modification of WO_3_ with RGO and phosphate. The oplimized 2RGO/WO_3_-3P produces the largest amount of ·OH species. The regularity of the detected ·OH amounts for the WO_3_-based photocatalysts is in agreement with that of the photocurrent densities and PEC activities. Therefore it’s could be further confirmed that the improved charge separation endows the excellent photocatalytic activities of modified WO_3_-based photocatalysts by producing more holes that induced larger amounts of ·OH.

To deepen the cognition on the improved photocatalytic activities of modified WO_3_ catalysts, the electrochemical impedance spectra (Nyquist plots) of WO_3_, 2RGO/WO_3_, WO_3_-3P and 2RGO/WO_3_-3P in dark (Figure S6D) and under light irradiation (Fig. 5D) were all collected to study the photogenerated charge transfer and separation. As observed for each sample the arc radius under light irradiation is smaller than that in dark, which is due to the decreased charge-transfer resistance. Moreover, under light irradiation the modified samples all exhibited smaller arc radius than WO_3_, among which 2RGO/WO_3_-3P showed the smallest the arc radius or rather the lowest charge-transport resistance. The decreased charge-transport resistance leads to a faster interfacial charge transfer^35^. Thus, it is deduced that after modification with RGO or phosphate both could improve charge separation by reducing the recombination of electron-hole pairs while the simultaneous modification of RGO and phosphate results in the best interfacial charge transfer, which is in accordance with its higher photocurrent response, PEC activities and amounts of ·OH.

Combining all the results above, the specific roles of the modifiers RGO and phosphate could be clarified, respectively. For the xRGO/WO_3_ samples, the increased photocurrent densities shown by the examined PEC activities and the decreased arc radius shown in EIS indicated the introduction of RGO did facilitate the photogenerated charge separation hence promote the activity of the WO_3_ in PEC reactions. Considering the favorable electrical conductivity of the RGO, for xRGO/WO_3_ the photogenerated electrons might transfer from WO_3_ to RGO through the heterojunction between RGO and WO_3_, which accounts for the improved photogenerated charge separation. Noteworthily, among all xRGO/WO_3_ samples, the optimum modfication amount for RGO exists, which is mainly because excessive amount of RGO would adsorb light resulting in cutting down the utilization of light. On the other aspect, similarly for phosphate modified WO_3_ catalysts the photogenerated charge separation was improved resulting in the enhanced activities in the PEC reactions. The optimum modification amount of phosphate also exists since over amount of phosphate would hinder the charge transfer.

**Figure 6:**
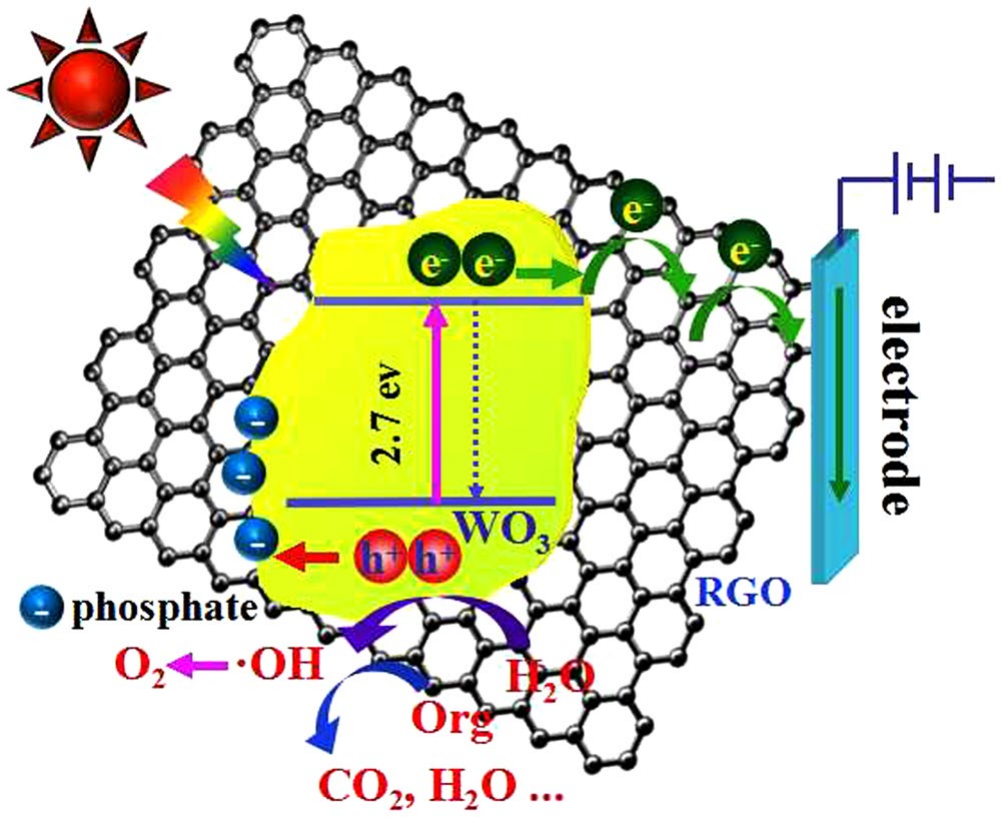
Schematic mechanism of WO_3_ co-modified with RGO and phosphate as a photoanode for PEC water oxidation to evolve O_2_ and pollutant degradation.

As our previous work, the phosphate would chemically absorb on the material surface resulting in forming the negative field^34^. In this work, the XPS P2p spectra have evidenced the chemically absorbance after the modification of phosphate. Moreover, the XPS W4f spectra for phosphate modified WO_3_ samples also proved the chemical interaction between introduced phosphate and WO_3_. Therefore rationally the photogenerated holes could be attracted by the formed negative fields originating from phosphate, which further facilitated the photogenerated charge separation. Hence the simultaneous modification of WO_3_ with RGO and phosphate facilitate the photogenerated charge separation on one hand by transferring the electrons to RGO on the other hand by inducing the holes transferring to WO_3_ surface. Therefore 2RGO/WO_3_-yP samples, especially 2RGO/WO_3_-3P with the most effective charge separation exhibited the best catalytic activities in the PEC reactions. Based on the analysis above the specific reaction mechanism with the co-modified WO_3_ with RGO and phosphate as the photocatalyst for the PEC water oxidation as well as pollutant degradation has been illustrated in Fig. 6. In specific the significantly facilitated photogenerated charge separation leads to more effective production of ·OH, which produces O_2_ in the PEC water oxidation and oxidizes the phenol to degrade to carbon oxide.

Herein, to realize the simultaneous modulation of photogenerated electrons and holes, we have successfully synthesized the WO_3_ co-modified by RGO and phosphate with the optimal amounts. Compared with pristine WO_3_, the PEC activities of co-modified WO_3_ for the water oxidation to evolve O_2_ and pollutant degradation have shown remarkable enhancement. The enhanced PEC activities are attributed to the improved charge separation endowed by the simultaneous modification of RGO and phosphate. Based on the evaluated hydroxyl radical amounts produced and the electrochemical impedance spectra, it is suggested that the improved PEC activities be attributed to the greatly enhanced photogenerated charge separation after chemical co-modification WO_3_ with RGO and phosphate groups. In specific, RGO assists to transfer electrons as the collectors and phosphate groups are capable of trapping holes via the formed negative field after phosphate disassociation. This work provides a feasible synthetic strategy to improve the PEC activities of nanosized WO_3_ for energy production and environmental remediation.

## Methods

All the reagents were of analytical grade and used as received without further purification. Deionized water was used throughout.

### Synthesis of materials

#### Synthesis of WO_3_ nanoplates

A modified phase-separated hydrolysis-solvothermal method was developed to synthesize WO_3_ nanoplates noted as WO_3_ for short with control by choosing WCl_6_ as tungsten resource and n-butanol as the organic phase. In the typical procedure, 10 mL of water and 8 mL of n-butanol containing 0.04 g of WCl_6_ were placed in Teflon-lined stainless autoclave. In which a 10 mL of weighing bottle is installed having *n*-butanol. The autoclave was kept at 120 °C for 15 hours. Then the autoclave was naturally cooled to room temperature, the WO_3_ is collected by separating from *n*-butanol. The WO_3_ was thoroughly rinsed with deionized water followed by absolute ethanol and dried under vacuum at 80 °C for 12 hours.

#### Preparation of RGO modified WO_3_

Graphene Oxide (GO) was prepared by an adjusted Hummers method^36^. Firstly a certain amount of GO was dissolved into 100 mL of deionized water with stirring for 60 min. And then 0.5 g of as-prepared WO_3_ powder was transferred into 20 mL ethanol, 20 mL GO solution was added dropwise to WO_3_/ethanol mixture with stirring for 15 min. Subsequently, the suspension was kept at 140 °C for 6 h in a Teflon-lined stainless-steel vessel to carry out hydrothermal reactions, and afterwards cooled naturally to room temperature. The as-prepared nanoparticles complexes were washed with deionized water and dried at 80 °C, followed by thermal treatment in air at 450 °C for 0.5 h, the typical powder was obtained, which is denoted as xRGO/WO_3_, where x represents the theoretical mass ratio percentage of RGO to WO_3_.

#### Preparation of phosphate modified WO_3_

To complete the modification with different amounts of phosphate, 0.5 g resulting WO_3_ powder was put into a certain content of phosphoric acid solution (20 mL). The mixture was kept under vigorous stirring at 80 °C until it is dried, and then calcined at 450 °C for 0.5 h. The phosphate-modified WO_3_ is denoted as WO_3_-yP, in which y represents the theoretical molar percent ratio of P to WO_3_.

#### Synthesis of WO_3_ co-modified with RGO and phosphate

According to the preliminary PEC activity test, among all xRGO/WO_3_ samples, 2RGO/WO_3_ has showed the best PEC activity, so the co-modification was performed to introduce phosphate to 2RGO/WO_3_. In specific by a simple wet chemical process, 0.5 g 2RGO/WO_3_ and 20 mL of phosphoric acid solutions with different concentration were kept under vigorous stirring at 80 °C until the water in the mixture was all evaporated. The resulted solid was then sintered at 450 °C for 0.5 h. The co-modified WO_3_ with RGO and phosphate is denoted as 2RGO/WO_3_-yP, in which y represents the theoretical molar percent ratio of P to WO_3_.

### Preparation of film electrodes

To fabricate the films for PEC measurements, corresponding pastes were prepared by our previous work^3^. For each sample 0.1 g powder was taken and dispersed in 1 mL isopropyl alcohol under vigorous magnetic stirring for 1 h. Then 0.05 g Macrogol-6000 was added to each sample and ultrasonically treated for 30 min, followed by continuous magnetic stirring for 1 h. At last, 0.01 mL acetyl acetone was added to the reaction mixtures and kept under vigorous magnetic stirring for 24 h.

The conductive fluorine doped tin oxide (FTO) coated glasses were cleaned by successive sonicate in detergent solution for 2 h and then washed with acetone cleaned FTO coated glasses were used as substrates for WO_3_, xRGO-WO_3_, WO_3_-yP and 2RGO/WO_3_-yP samples. Thin films of all samples were prepared by doctor blade method using Scotch tape as a spacer. The prepared films were dried in air for 30 min and then calcined at 450 °C for 30 min. The FTO coated glasses with thin film were cut into 1.0 cm × 3.0 cm pieces having film surface area 1.0 cm × 1.0 cm to use for a photoanode material.

### Characterization of materials

The XRD patterns of the material was characterized with the help of a Bruker D8 Advance X-ray diffractometer equipped with a graphite monochromatized Cu Kα radiation (γ = 1.541874 Å). The UV-vis DRS of the samples were measured with a Model Shimadzu UV2550 spectrophotometer. Scanning electron microscopy (SEM) images of films were taken using a Hitachi S-4800 instrument operating at 15 KV. The compositions and elemental chemical states of the samples were examined through X-ray photoelectron spectroscopy (XPS) using a Kratos-Axis Ultra DLD apparatus with an Al (mono) X-ray source. The binding energies mentioned were calibrated with respect to the signal for adventitious carbon (binding energy = 284.6 eV). Raman spectra were recorded on a Jobin Yvon HR800 micro-Raman spectrometer with 457.9 nm laser.

### Photoelectrochemical measurements

A typical three-electrode configuration was used to measure the PEC properties in the preparation process. The as-prepared films, platinum foil, and Ag/AgCl (saturated KCl) were used as the working electrode, counter electrode, and reference electrode, respectively. Photocurrents were measured in 0.5 M Na_2_SO_4_ using a commercial computer controlled potentiostat (AUTOLAB PG STAT 101). The photocatalytic electrodes were irradiated with light from a 150 W xenon lamp.

To measure the amount of O_2_ produced in the PEC water oxidation, the as-prepared films were used as working electrodes in a sealed quartz cell with 100 mL of 0.5 M Na_2_SO_4_ and oxygen-free nitrogen gas was employed to bubble through the electrolyte before the experiment. The films were illuminated from the FTO glass side, the effective area of the working electrode was about 0.5 cm^2^, at the constant bias of 0.6 V. During the experiment, the amount of O_2_ produced was detected quantitatively with an Ocean Optics fluorescence-based oxygen sensor (NFSC 0058) by putting the needle probe into the electrolyte, near to the working electrode, and the irradiation was lasted for 500 s using 150 W xenon lamp.

Phenol (10 mg/L) was used as the model pollutants to evaluate the PEC degradation. A 150 W xenon lamp was utilized as the light source, using 0.5 M Na_2_SO_4_ as the electrolyte. Before light irradiation, the fabricated 2.0 × 2.5 cm^2^ samples were immersed in the mixed solutions containing phenol reacted with catalyst electrode for 30 min under dark conditions to establish an adsorption/desorption equilibrium. The concentrations of phenol and its degradation intermediates were analyzed by a UV-vis spectrophotometer (Shimadzu UV2550). The ratio of phenol concentrations C/C_0_ could be calculated using C/C_0_ = A/A_0_, where C_0_ and C are the concentrations of the phenol solution at irradiation time 0 and t, and A_0_ and A are the corresponding absorbance values at 270 nm. For comparison, the photocatalytic degradation experiment was also performed by using the same test system without applying an external potential.

### Evaluation of produced ·OH amount

Hydroxyl radical measurement was carried out in 0.001 M coumarin aqueous solution in a quartz reactor of 40 mL containing 0.05 g of sample powder. Prior to irradiation, the reactor was magnetically stirred for 10 min to attain an adsorption-desorption equilibrium. After irradiation for 1 h, the sample was centrifuged and a certain amount was transferred into a Pyrex glass cell for the fluorescence measurement of 7-hydroxycoumarin at 390 nm excitation wavelength and emission wavelength at 460 nm through a spectrofluorometer (Perkin-Elmer LS55)^37^.

### Electrochemical impedance spectroscopy measurement

The electrochemical impedance spectroscopy (EIS) was performed using a three-electrode configuration with the Prinston Applied Research Versa STAT 3 and carried out over the frequency range from 102 to 105 Hz with by applying sinusoidal perturbations of 10 mV with a bias of +0.6 V in a 0.5 M Na_2_SO_4_ solution.

## Electronic supplementary material

Supplementary Information
