# Supplementary material for: Enhanced photoelectrochemical activities for water oxidation and phenol degradation on WO3 nanoplates by transferring electrons and trapping holes
Source: Sci Rep. 2017 May 2;7:1303. doi: 10.1038/s41598-017-01300-7 (PMC5430972; doi:10.1038/s41598-017-01300-7)
Supplement: Supplementary file 1 — Supplementary Information [file 41598_2017_1300_MOESM1_ESM.pdf]

Supplementary Information

Enhanced photoelectrochemical activities for water oxidation and phenol degradation on WO_3 nanoplates by transferring electrons and trapping holes

Liqun Sun[a,b], Yuying Wang[a], Fazal Raziq[a], Yang Qu[a], Linlu Bai[a,c,]*, and Liqiang Jing[a,]*

[a]Key Laboratory of Functional Inorganic Materials Chemistry (Heilongjiang University), Ministry of Education, International Joint Research Center for Catalytic Technology, School of Chemistry and Materials Science, Harbin 150080, P. R. China.

Fax: (+86)-451-86604760

E-mail: jinglq@hlju.edu.cn

[b]College of Chemical Engineering, Daqing Normal University, Key Laboratory of Oilfield Applied chemistry, College of Heilongjiang Province, Daqing 163712, P. R. China.

[c]College of Chemical and Environmental Engineering, Harbin University of Science and Technology, Harbin 150040, P. R. China.

E-mail: lbai2@e.ntu.edu.sg

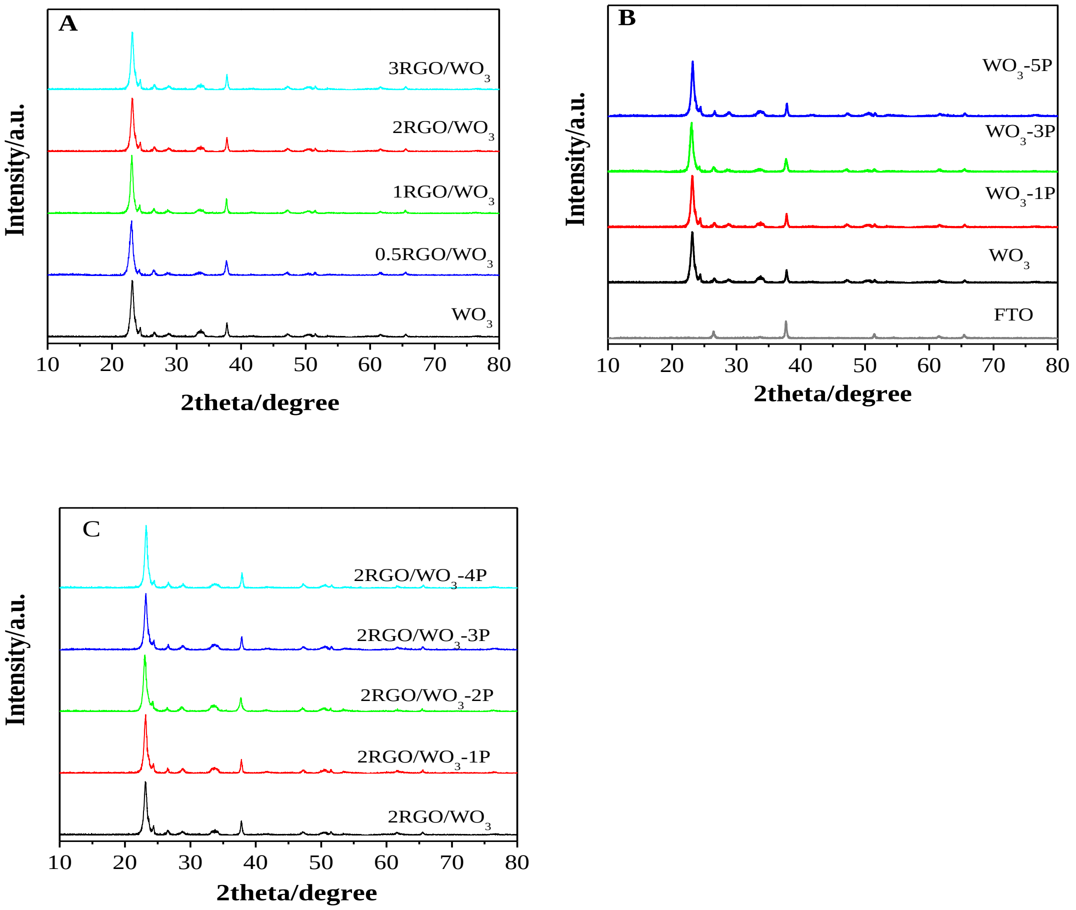

Fig. S1 XRD patterns of FTO, WO_3 film and xRGO/WO_3 films (A) WO_3-yP (B) and 2RGO/WO_3-yP (C).

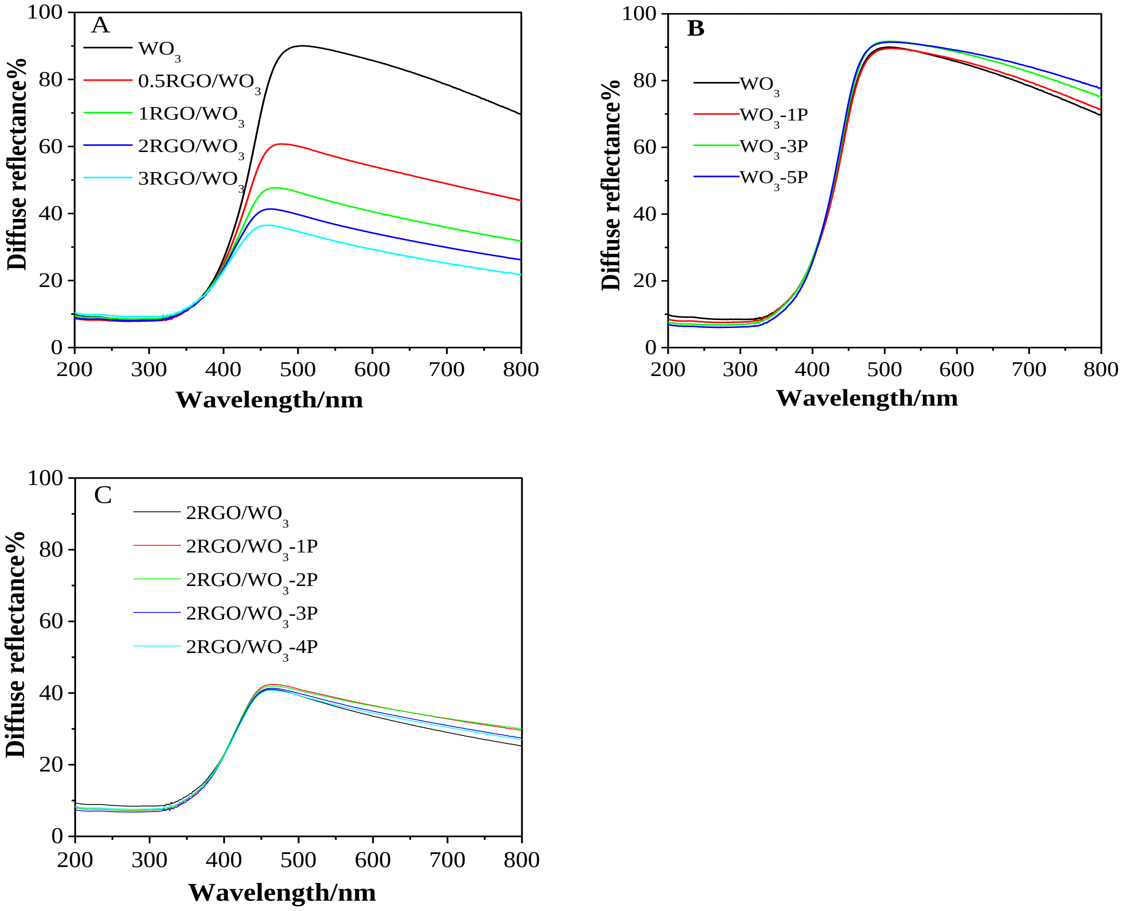

Fig. S2 UV-*vis* diffuse reflectance spectra of WO_3, xRGO/WO_3 (A), WO_3-yP (B) and 2RGO/WO_3-yP (C).

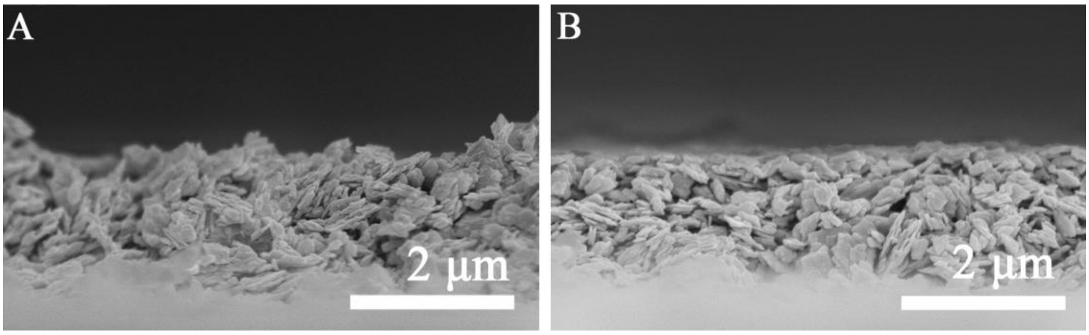

Fig. S3 Side-viewed SEM images of WO_3 (A) and 2RGO/WO_3-3P (B) films.

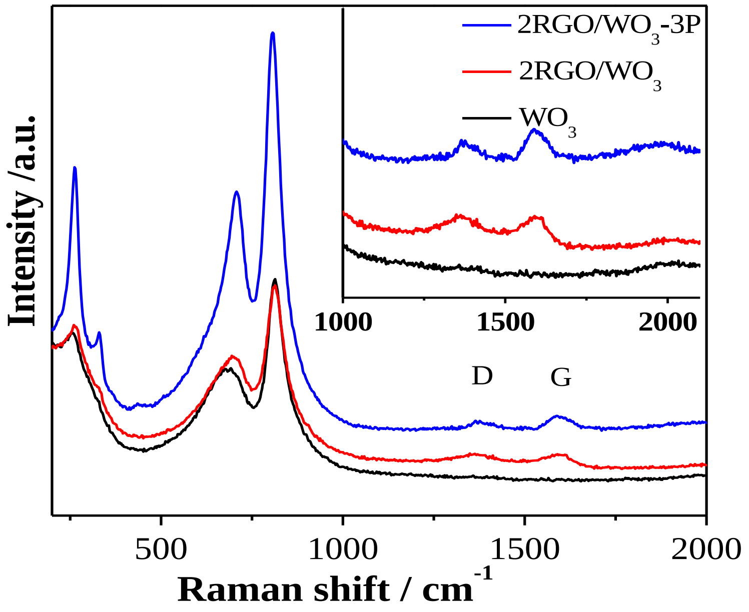

Fig. S4 Raman spectra of WO_3, $2RGO/WO_3$ and $2RGO/WO_3$-3P.

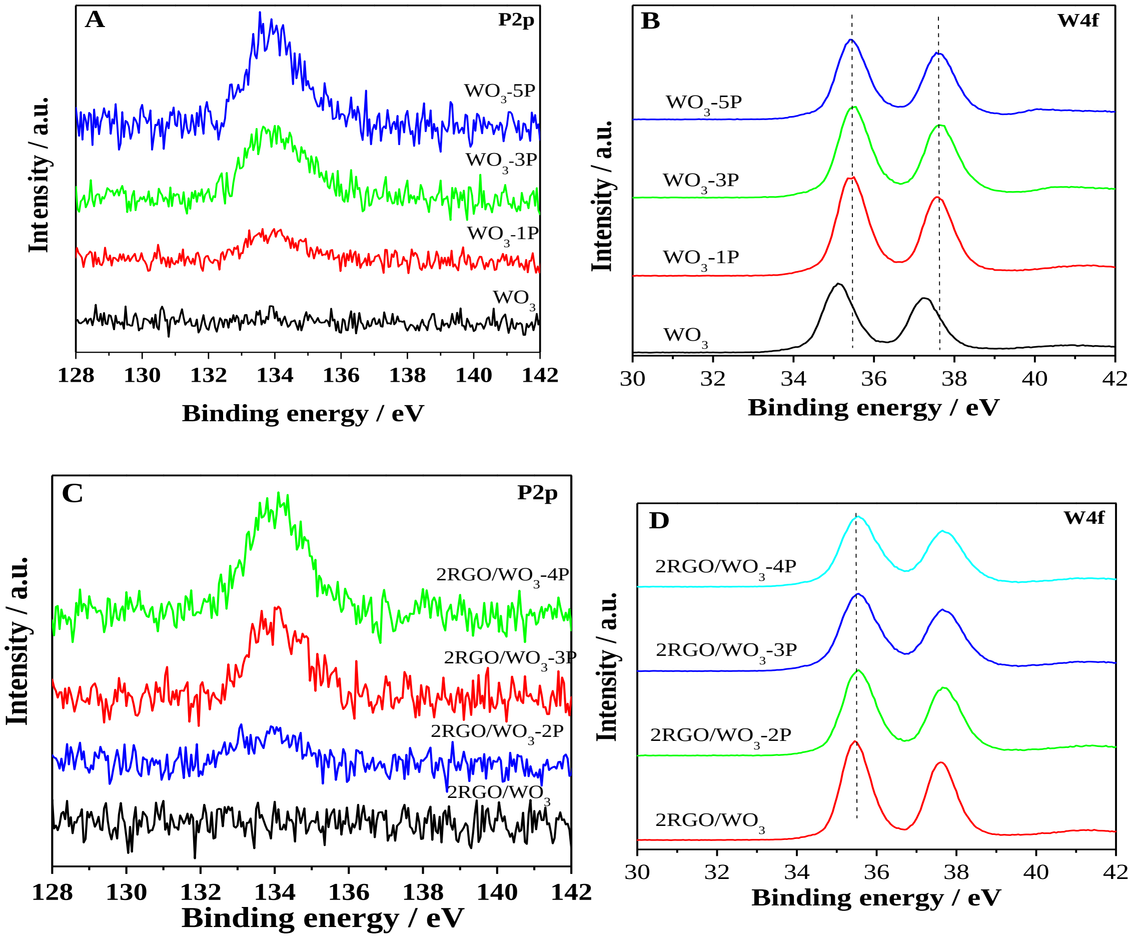

Fig. S5 P2p XPS spectra (A) and W4f XPS spectra (B) of WO_3-yP; P2p XPS spectra (C) and W4f XPS spectra (D) of 2RGO/WO_3-yP.

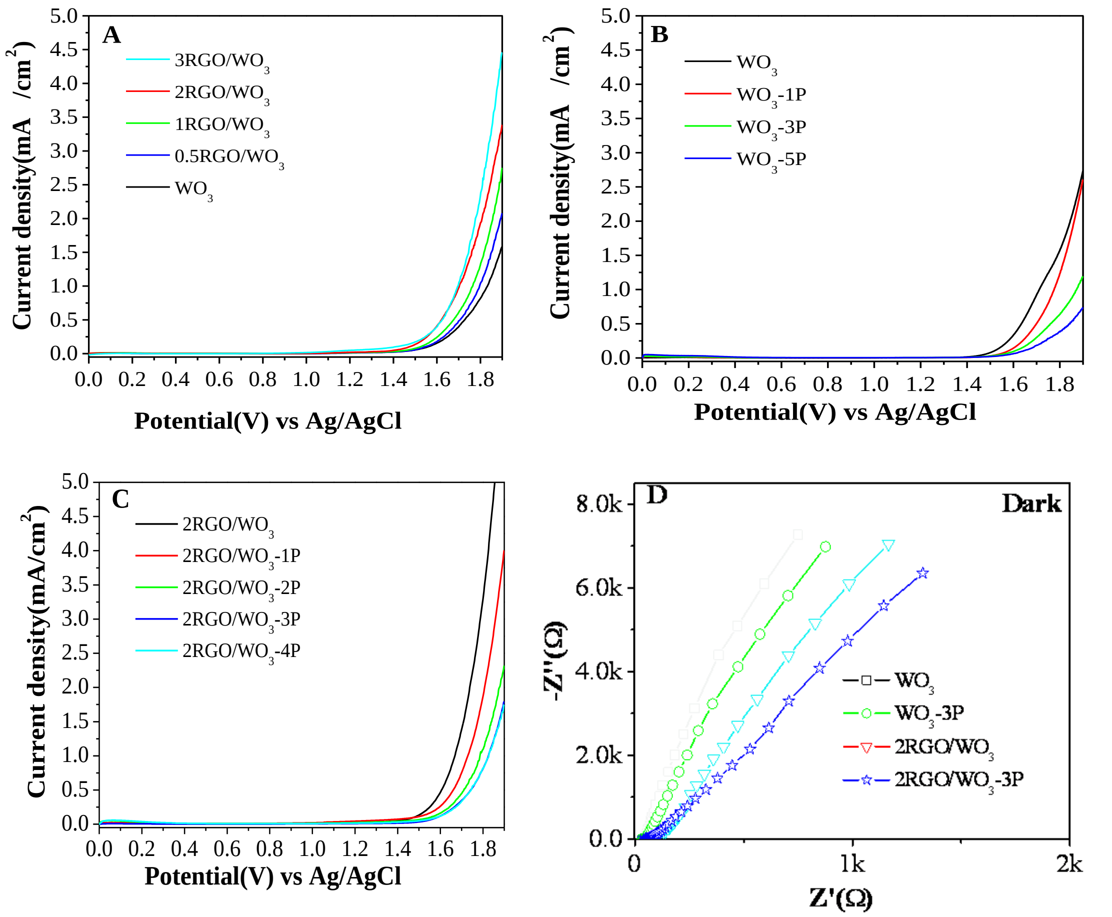

Fig. S6 I-V curves of WO_3 and xRGO/WO_3 (A), WO_3-yP (B) and 2RGO/WO_3-zP (C) in dark; electrochemical impedance spectra in dark of WO_3, WO_3-3P, 2RGO/WO_3 and 2RGO/WO_3-3P (D). Potentials were measured in 0.5M Na_2SO_4 electrolyte solution. A three-electrode cell was used with the testing film as the working electrode, Ag/AgCl (saturated KCl solution) as the reference electrode, and Pt plate as the counter electrode.
